# Effects of the Water Matrix on the Degradation of Micropollutants by a Photocatalytic Ceramic Membrane

**DOI:** 10.3390/membranes12101004

**Published:** 2022-10-16

**Authors:** Shuyana A. Heredia Deba, Bas A. Wols, Doekle R. Yntema, Rob G. H. Lammertink

**Affiliations:** 1 Wetsus European Center of Excellence for Sustainable Water Technology, 8911 MA Leeuwarden, The Netherlands; 2 Membrane Science and Technology, Faculty of Science and Technology, University of Twente, Drienerlolaan 5, 7522 NB Enschede, The Netherlands; 3 KWR Watercycle Research Institute, 3430 BB Nieuwegein, The Netherlands

**Keywords:** photocatalytic membrane, TiO_2_, micropollutant degradation, mixture and water matrix effect, AOP, water treatment

## Abstract

The consumption of pharmaceuticals has increased the presence of micropollutants (MPs) in the environment. The removal and degradation of pharmaceutical mixtures in different water matrices are thus of significant importance. The photocatalytic degradation of four micropollutants—diclofenac (DCF), iopamidol (INN), methylene blue (MB), and metoprolol (MTP)—have been analyzed in this study by using a photocatalytic ceramic membrane. We experimentally analyzed the degradation rate by using several water matrices by changing the feed composition of micropollutants in the mixture (from mg· L${}^{-1}$ to μg·L${}^{-1}$), adding different concentrations of inorganic compounds (NaHCO_3_ and NaCl), and by using tap water. A maximum degradation of 97% for DCF and MTP, and 85% for INN was observed in a micropollutants (MPs) mixture in tap water at environmentally relevant feed concentrations [1–6 μg·L${}^{-1}$]${}_{o};$ and 86% for MB in an MPs mixture [1–3 mg·L${}^{-1}$]${}_{o}$ with 100 mg·L${}^{-1}$ of NaCl. This work provides further insights into the applicability of photocatalytic membranes and illustrates the importance of the water matrix to the photocatalytic degradation of micropollutants.

## 1. Introduction

Medicines are consumed daily with and without prescriptions, and some of these consumed pharmaceuticals are not metabolized and are eventually discharged into sewers [1]. These then become part of the organic contaminants—also known as micropollutants (MPs)—which pose a significant challenge for conventional wastewater treatment plants, which currently lack efficient methods to completely remove these substances [2]. Available abiotic (physical–chemical) methods usually do not alter the chemical structure of the MPs but simply transfer them to a different phase which requires a secondary treatment. Biotic processes with microorganisms have proven to be insufficient, as not all micropollutants are completely removed [3]. As a result, MPs are frequently detected in environmental matrices such as surface water, and their long-term ecotoxicological effect is as yet unknown [4].

To achieve better and more consistent micropollutant (MP) removal, several advanced treatment methods are available, including advanced oxidation processes (AOPs), activated carbon adsorption, membrane bioreactors, nanofiltration, and reverse osmosis [5]. Combining one or more conventional/advanced treatments could offer a solution to achieve further removal of pharmaceutical contaminants [6]. One promising combination for water treatment concerns an advanced oxidation process (AOP) like photocatalytic oxidation and membrane separation [7]. Photocatalytic oxidation with titanium dioxide has received particular attention due to the nonspecific nature of reactive oxygen species (ROS) produced under UV irradiation. Membrane separation processes are increasingly used, as they rely on a physical separation (usually with no addition of chemicals in the feed stream) and have many advantages, including easy operation and high separation efficiency. Evidently, the retentate of membrane separation processes still requires adequate treatment.

Membrane retention and photocatalytic oxidation can be combined by coating membranes with titanium dioxide (TiO_2_) particles, thus creating photocatalytic membranes. The TiO_2_ particles require the activation by a light source with photon energy greater than the band gap, which can excite an electron from the valence band to the conduction band, e${}^{-}$, and leave an electron hole, h${}^{+}$, in the valence band [8,9]. These energy carriers (e${}^{-}$ and h${}^{+}$), in contact with oxygen and water, generate ROS that can attack organic molecules. Moreover, the electron-hole pairs can also react directly with the pollutants [9]. A downside of this process is the recombination of e${}^{-}$ and h${}^{+}$ pairs in the absence of scavengers, releasing heat instead of oxidizing the organic compounds. A photocatalytic membrane may provide another good option for removing organic MPs in the environment as a part of the wastewater-treatment process.

Studies on the functionality of photocatalytic membranes to degrade co-existing pharmaceuticals or other chemicals are needed, as the synergistic effect of these mixtures brings more complex toxicity to living organisms, which is challenging to forecast and resolve [10]. Only a few recent studies concern the use of titanium dioxide-coated membranes to degrade MP mixtures. These studies were primarily carried out with target MPs in pure water, with less attention to the water matrix. Some examples include Fernández et al. [11], who studied the photocatalytic degradation of 33 trace organic contaminants in a submerged membrane photocatalysis reactor, Hu et al. [12], who degraded a suite of 13 medicines with TiO^2^ nanowire membranes, Arlos et al. [13], who assessed the treatment of 10 pharmaceuticals and personal care products with different isoelectric points on porous TiO_2_ supports, and Lofti et al. [14], who used nanoparticles in a nanoporous membrane for the removal of four steroid hormones. There is a lack of studies that consider the effect of water matrices in the degradation of MP mixtures by photocatalytic membranes, and this study provides an important step toward implementing photocatalytic pharmaceutical degradation on an industrial scale.

In the present study, the applicability of photocatalytic membranes to degrade pharmaceuticals in different water matrices is investigated by changing the feed concentration of micropollutants in the mixture (from mg·L${}^{-1}$ to μg·L${}^{-1}$), adding different concentrations of inorganic compounds (NaHCO_3_ and NaCl), and testing the mixture in tap water. From the vast number of pharmaceutical compounds in the environment, our study focused on the photocatalytic degradation of four medicines found in surface waters at up to μg·L${}^{-1}$ levels. These include diclofenac (DCF), a widely prescribed nonsteroidal anti-inflammatory drug, iopamidol (INN), a popular contrast agent used in medical imaging, methylene blue (MB) which is increasingly used in various medical fields as a dye and photosensitizer [15], and metoprolol (MTP), a β blocker widely used in both hospitals and households to lower blood pressure, slow the heart rate, and decrease the oxygen demand of the heart. The removal efficiency of these compounds provides a relevant and realistic challenge in current water-treatment processes.

## 2. Materials and Methods

### 2.1. Materials

Diclofenac (DCF) C_14_H_10_Cl_2_NNaO_2_ (CAS 15307-79-6), iopamidol (INN) C_17_H_22_I_3_N_3_O_8_ (CAS 60166-93-0), and metoprolol tartrate salt (MTP) (C_15_H_25_NO_3_)_2_C_4_H_6_O_6_ (CAS 56392-17-7) were purchased from Sigma Aldrich (Germany). Methylene blue (MB) C_16_H_18_ClN_3_S (CAS 61-73-4) was acquired from Boom BV (Meppel, The Netherlands), and sodium sulfate anhydrous Na_2_SO_4_ (CAS 7757-82-6), sodium hydrogen carbonate NaHCO_3_ (CAS 144-55-8), and sodium chloride NaCl (CAS 7647-14-5) were obtained from VWR chemicals (Leuven, Belgium). All were used as received. Several stock aqueous solutions at concentrations of 20 mg·L${}^{-1}$ DCF, 10 mg·L${}^{-1}$ INN, 32 mg·L${}^{-1}$ MB, 10 mg·L${}^{-1}$ MTP, 4261 mg·L${}^{-1}$ sodium sulfate, and 700 mg·L${}^{-1}$ sodium bicarbonate were prepared. Ultrapure water from a Milli-Q Advantage A10 system (Merck Millipore, Darmstadt, Germany) was used for the preparation of the stock and feed solutions. The tap water (TW) was collected from the local drinking water supply (Vitens, Leeuwarden The Netherlands).

### 2.2. Photocatalytic Degradation Experiments

Dead-end filtration and degradation experiments were performed in a custom photocatalytic membrane reactor (PMR) consisting of a TiO_2_-coated alumina membrane within a PMMA holder, placed inside a cupboard to protect the micropollutants from the reaction with ambient light (see Heredia Deba et al. [16] for details on the setup configuration and the PMR). For the cross-flow experiments, a stainless steel PMR with the same structure but without a feed reservoir was employed. In the stainless steel module, the space on top of the membrane is limited by the o-ring (EPDM 25 × 1.5, Eriks) thickness after closing the module.

For the experiments with the MPs independently (singles), the aqueous solutions were pumped into the setup with fluxes in the nanofiltration range, 1.6, 3.3, 6.5, 9.7, 13.0, and 16.2 L·m${}^{-2}$·h${}^{-1}$ and additionally for DCF 19.5 and 21.1 L·m${}^{-2}$·h${}^{-1}$; these experiments were repeated three times to analyze the reproducibility of results. For the experiments with the mixtures, three fluxes (1.6, 6.5, and 16.2 L·m${}^{-2}$·h${}^{-1}$) and two repetitions were investigated, as the reproducibility of the experiments was high.

The feed concentration varied across experiments and micropollutants. For the experiments with the single MP and for those in a mixture, the initial concentration was 2 mg·L${}^{-1}$ DCF, 1 mg·L${}^{-1}$ INN, 4 mg·L${}^{-1}$ MB, and 1 mg·L${}^{-1}$ MTP (molar concentration [μmol·L${}^{-1}$] ratio 6.8:1.3:12.5:3.7 DCF:INN:MB:MTP). For the experiments with low concentrations (LC) the feed was 2 μg·L${}^{-1}$ DCF, 6 μg·L${}^{-1}$ INN (3 μg·L${}^{-1}$ in TW), 4 μg·L${}^{-1}$ MB, and 2.5 μg·L${}^{-1}$ MTP (molar concentration [nmol·L${}^{-1}$] ratio 6.8:7.7(3.9):10:9.4 DCF:INN:MB:MTP). For experiments regarding the effect of background water constituents, the initial concentration in the mixture was 45 mg·L${}^{-1}$ (0.7 mM) and 215 mg·L${}^{-1}$ (3.5 mM) of bicarbonate ions, and 61 mg·L${}^{-1}$ (1.7 mM), and 607 mg·L${}^{-1}$ (17.1 mM) of chloride ions. A concentration of 142 mg·L${}^{-1}$ (1 mM) of sodium sulfate was used as background in all the singles experiments and the experiments with bicarbonate to avoid corrosion in the system. For the experiments with sodium chloride and low concentrations of micropollutants, sodium bicarbonate with a concentration of 23.4 mg·L${}^{-1}$ (0.3 mM) was used. In the experiments with TW, no extra background was added. The chemical composition of the mixture in TW can be found in Appendix A. The natural pH of the system was used without further adjustment. For the experiments with sodium sulfate in the background, the pH was between 6 and 7, except for the experiments with bicarbonate, in which the pH was approximately 8. For the experiments with bicarbonate in the background, the pH was between 7 and 7.5. In the experiments with tap water, the pH was 8. A table summarizing the measured feed solution and permeate pH can be found in Appendix B.

Two photocatalytic membranes were used for the experiments named A and B. Membrane A was utilized for the singles experiments, and membrane B for the experiments with the mixtures. In order to compare the photocatalytic properties of both membranes, the experiments with MB and MTP were reproduced, and the data confirmed that the performance of both membranes was similar. More data about the membrane fabrication can be found in our previous work [16], as we used the same titanium dioxide suspension (Evonik, VP Disp. W 2730 X) and deposition technique (dip coating). Results on the morphology of membranes A and B can be found in Appendix C.

Before each experiment, the membranes are equilibrated with the feed solution for at least 120 min at 16.2 L·m${}^{-2}$·h${}^{-1}$ to ensure adsorption equilibrium before the degradation measurements. This is because the pre-adsorption of reactants on the surface of the TiO^2^ membrane may lead to a more efficient electron-transfer process [17,18]. After equilibrating, the LED was turned on, and samples were taken from the permeate every 30 min, except for MB, which was continuously monitored. The experiments were finalized when the outlet concentration reached a steady value for each filtration rate.

The input radiation level was set before each run to 210 W·m${}^{-2}$ and measured by using a power meter (Thorlabs) with a thermal power sensor head (S310-C). Control experiments with MB were carried out with a membrane without the TiO_2_ layer to rule out effects other than photocatalytic oxidation, e.g., bulk photolysis. It should be noted that none of the used MPs absorb photons in the used wavelength ($\lambda_{max}$ = 366 nm) (Appendix D), and hence no direct photolysis of the MPs is expected to take place in our system (Grotthuss–Draper law). Photolysis of DCF is generally reported in studies utilizing direct sunlight [19,20] or lamps emitting polychromatic light, including those using filters restricting the transmission of wavelengths below 290 nm [21,22]. The overlap with the absorption spectrum of DCF ($\lambda_{max}$ = 194 nm) with a shoulder absorbance of up to ∼320 nm could explain this effect. Martínez et al. [23] reported photolysis upon near-UV-Vis irradiation (mainly at 366 nm), and Calza et al. [24], who used a xenon arc lamp and special glass filter to restrict the emissions below 290 nm, and Rizzo et al. [25], who used a black light fluorescent lamp emitting radiation between 300 and 420 nm, did not report any significant DCF degradation via photolysis.

### 2.3. Analytical Methods

The presence of the initial reactants in the permeate was measured without considering the intermediate products. Detection and quantification of DCF, INN, and MTP were performed by using an Agilent LC-MS/MS system consisting of Agilent infinity 1260 LC-system (degasser, binary pump, autosampler with cooler tray, and column oven) and Agilent 6420 triple quadrupole mass spectrometer with an electrospray ion source. The samples (injection volume 5 μL) were separated by using an Agilent Zorbax Eclipse plus C18 RRHD (50 × 2.1 mm, particle size 1.8 μm) and eluted with a mixture of ammonium formate buffer in water and acetonitrile. The compounds were detected and quantified on the 6420-QQQ-MS by using compound-specific multiple dynamic MRM transitions. Three methods were used to analyse the MPs to avoid the interference of the ions from the salts in the measurements. Method A, an isocratic method using 60:40 buffer:acetonitrile, was used to analyze DCF and MTP when studied independently. Method B, an isocratic method using 19:1 buffer:acetonitrile, was applied to detect INN for the experiments independently. Method C, a gradient program of acetonitrile (5 to 95%) and buffer, was used for all micropollutants in the experiments in the mixtures.

The discoloration of MB was continuously monitored by passing the permeate through a flow cell (FIA-Z-SMA-ML-PE flow cell, 10 mm path length) connected to a UV-VIS spectrometer (flame model spectrometer with sony detector, Ocean Optics). The monitored wavelength was 664 nm, corresponding to the maximum absorption peak of MB. Method C was also used to quantify the MB concentration in the LC-MS/MS, especially with the experiments with the MPs at low concentrations, as these are outside of the spectrometer measurement range. We observed that the MB concentrations measured by the LCMS/MS varied with the sample preparation time. MB can act as a photosensitizer, as it has a strong light absorption in the visible range. The samples were diluted in transparent flasks, exposed to the laboratory ambient light, and decomposed during the time the solution was in the flask. Furthermore, we discovered that for low concentrations, we could measure the desorption of MB from the PMR (o-rings, glue, and tubing, saturated with methylene blue from previous experiments with high MB concentrations) because the permeated MB concentration was reported higher than in the feed. Therefore, those results are not reported in this manuscript.

### 2.4. 1D Transport and Surface Reaction Model and Diffusion Coefficient

A simple 1D transport and surface reaction model was applied to analyze the experimental results. Details about the model can be found elsewhere [16]. This model is based on a convection-diffusion equation, with a constant inlet concentration and a surface reaction as corresponding boundary conditions. The solution of the ODE with the abovementioned boundary conditions for the permeate concentration is given as

(1) $$c_{p}=\alpha c_{m}=\frac{\alpha e^{\mathrm{Pe}}\mathrm{Pe}}{\mathrm{Pe}\left(1-\alpha \right)-\mathrm{Da}{}_{II}+e^{\mathrm{Pe}}\left(\alpha \mathrm{Pe}+\mathrm{Da}{}_{II}\right)},$$

where three different dimensionless numbers are used. Pe represents the Péclet number, Pe =uL/D, with linear velocity *u* [m·s${}^{-1}$], liquid reservoir height *L* [m], and diffusivity *D* [m${}^{2}$·s${}^{-1}$], Da${}_{II}$ is the second Damköhler number, Da${}_{II}$ $=\;k^{\prime }L/D$, with surface reaction rate constant $k^{\prime }$ [m·s${}^{-1}$], and α which shows the membrane function and is the ratio of permeate concentration to concentration at the membrane $\left(c_{p}/c_{m}\right)$.

The diffusion coefficient values for MB and DCF used in this investigation were taken from the literature. Meanwhile, the experimental diffusion coefficients of MTP and INN were not found in the literature, so the values used in this study were estimated by using the Wilke–Change correlation [26] given by

(2) $$D_{e}=7.4\times 10^{-8}\frac{{\left(\varphi M_{W}\right)}^{0.5}T}{\mu V_{M}^{0.6}},$$

where φ defines the association parameter with the solvent (set to 2.6 for water), $M_{W}$ the molar mass of water (g·mol${}^{-1}$), *T* the temperature (K), μ the water viscosity (cP), and $V_{M}$ the molar volume of the solute (cm${}^{3}$·mol${}^{-1}$). The molar volumes were calculated by relating the Van der Waals volume obtained from the molecular software PaDeL [27] to the LeBas volume by $V{}_{M}$ = 1.06$V{}_{W}$ [28].

The diffusion coefficient values form the literature, $D_{t}$, and the estimations using the Wilke–Change correlation, $D_{e}$ can be found in Table 1. The estimated values are in the range of previously reported values.

## 3. Results and Discussion

### 3.1. Membrane Retention

The intrinsic membrane retention is represented by 1-α (Equation (1)) in the 1D transport and surface reaction model. To calculate the value of α, cross-flow experiments with iopamidol (the biggest molecule among the MPs used in these experiments) and methylene blue were carried out with fluxes of 16.2, 32.5, 48.7, and 65.0 L·m${}^{-2}$·h${}^{-1}$. No significant differences were detected in the retentate and permeate concentrations for the tested flows; therefore, we justify using α = 1 in the 1D model, referring to no retention by the membrane ($c_{m}=c_{p}$). The equation to fit the permeate concentration versus Pe then only contains one fitting parameter, Da${}_{II}$:

(3) $$c_{p}=\frac{e^{\mathrm{Pe}}\mathrm{Pe}}{e^{\mathrm{Pe}}\left(\mathrm{Pe}+\mathrm{Da}{}_{II}\right)-\mathrm{Da}{}_{II}}.$$

### 3.2. MPs Degradation and Transport and Surface Reaction Model

**Figure 1:**
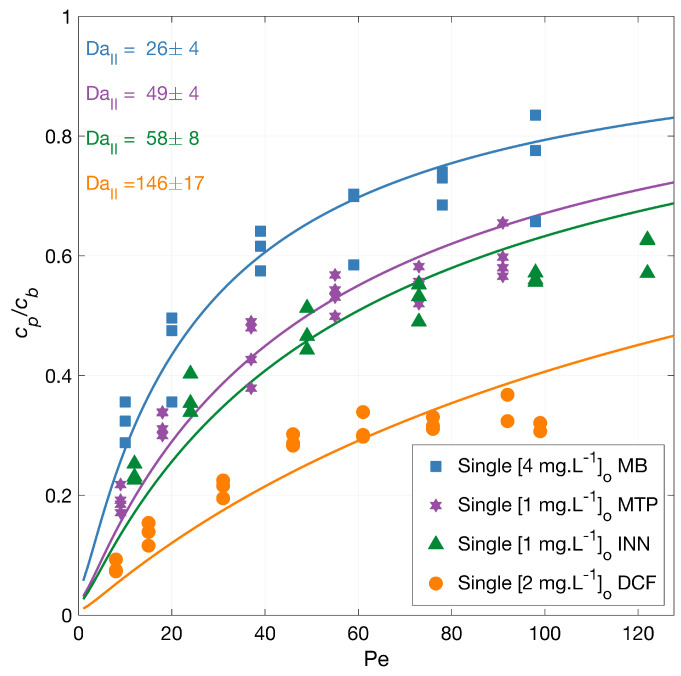
MPs degradation vs. filtration rate. Symbols depict experimental data, and the lines correspond to the mass transport and surface reaction model with indicated fitted Da${}_{II}$ number. The experiments were carried out with the micropollutants independently, and the legend shows the feed concentrations [$c_{b}$]${}_{o}$. Conditions: 366 nm radiation, 210 W·m${}^{-2}$, 1.6, 3.3, 6.5, 9.7, 13.0, and 16.2 L·m${}^{-2}$·h${}^{-1}$ and additionally for DCF 19.5 and 21.1 L.m${}^{-2}$·h${}^{-1}$, and 142 mg·L${}^{-1}$Na_2_SO_4_.

Degradation experiments with diclofenac (DCF), iopamidol (INN), methylene blue (MB), and metoprolol (MTP) independently (singles) were conducted in a single-pass dead-end PMR under an average irradiation intensity of 210 W·m${}^{-2}$. The normalized permeate concentration ($c{}_{p}$/$c_{b}$) as a function of the Péclet number (Pe = uL/D) is plotted together with model fits and shown in Figure 1.

The lines in Figure 1 represent the mass transport and surface reaction model fits. The symbols represent the experimental results for the MP degradation to which the model is fitted. The fits are represented by the corresponding Da${}_{II}$ numbers that are indicated in the figure accompanied with their 95% confidence intervals. Higher flow rates result in lower contact time with the radicals for the reaction to occur, and thus an increased outlet concentration. A higher second Damköhler number (Da${}_{II}$) represents a faster reaction because this value presents the surface reaction to mass transport rate. Under the same conditions, the order in the overall degradation from higher to lower was DCF > INN > MTP > MB, with a similar Damköhler value for INN and MTP. The degradation degree at the lowest flux was 92% for DCF, 76% for INN, 68% for MB, and 81% for MTP.

Table 2 shows a summary of physicochemical properties and reaction constants for the MPs used in our experiments. It is important to look at these reaction constants only as a reference point because those values, and the concentration of formed radicals or electrons have not been measured for our experimental conditions. In addition, the initial molar concentration varied between micropollutants in a molar ratio of 6.8:1.3:3.7:12.5 DCF:INN:MTP:MB, and for a system with the same amount of radicals generated and not added scavengers, the compounds with larger molar concentration need more radicals to be degraded.

Hydroxyl radicals are considered the primary oxidant in the photocatalytic process [8,31]. The hydroxyl reaction rate constants in Table 2 indicate that MB is the most reactive with hydroxyl radicals, followed by DCF, MTP, and finally INN, but this order was not observed during our photocatalytic experiments. Buxton et al. [32] reported that the order of magnitude for $k_{OH}$ for most reactants with hydroxyl radical ranges between 10${}^{8}$ and 10${}^{10}$ L·mol${}^{-1}$·s${}^{-1}$, which demonstrates the relatively nonselective nature of OH^−^ radical reactions in aqueous solution [33]. At the same time, in Table 2, the reaction rate constants with solvated electrons point at INN as the fastest reacting with e${}^{-}$ followed by MB, DCF, and MTP. This order was not observed in our experiments either.

The pH of the solution and the charge of the MP also play an important role during the photocatalytic degradation. The point of zero charge (pzc) for TiO_2_ is between pH 4.5 and 7.0, depending on the type and composition of the photocatalyst [34,35]. The titania surface groups, TiOH, can protonate or de-protonate according to

$$\mathrm{TiOH}+H^{+}↔{\mathrm{TiO}H_{2}}^{+}$$

$$\mathrm{TiOH}+{\mathrm{OH}}^{-}↔{\mathrm{TiO}}^{-}+H_{2}O.$$

During the experiments with the MPs independently, the pH was between 6 and 8.5—a negative surface membrane charge, which should favor the adsorption of positively charged molecules.

**Table 2:** Summary of relevant physicochemical properties of the selected MPs.

MP Abbr.	k${}_{\mathrm{OH}}$ [10${}^{9}$ L·mol${}^{-1}$·s${}^{-1}$]	k${}_{e^{-}\mathrm{aq}}$ [10${}^{9}$ L·mol${}^{-1}$·s${}^{-1}$]	pK${}_{a1}$, pK${}_{a2}$	Charge at pH > 6
DCF	9.29 ± 0.09 [36]	1.53 ± 0.03 [36]	4.15 [37]	negative
INN	3.42 ± 0.28 [38]	33.7± 0.5 [38]	10.7 [39]	positive
MB	11 [40]	25 [32]	3.14 [41]	positive
MTP	8.39 ± 0.06 [42]	0.173 ± 0.003 [42]	9.67, 14.09 [43]	positive

MP Abbr. = Micropollutant abbreviation, k${}_{\mathrm{OH}}$ [L·mol${}^{-1}$·s${}^{-1}$], hydroxyl reaction rate constant, k${}_{e^{-}\mathrm{aq}}$ [L·mol${}^{-1}$·s${}^{-1}$], hydrated electron reaction rate constant, pK_*a*_, acid dissociation constant.

**Figure 2:**
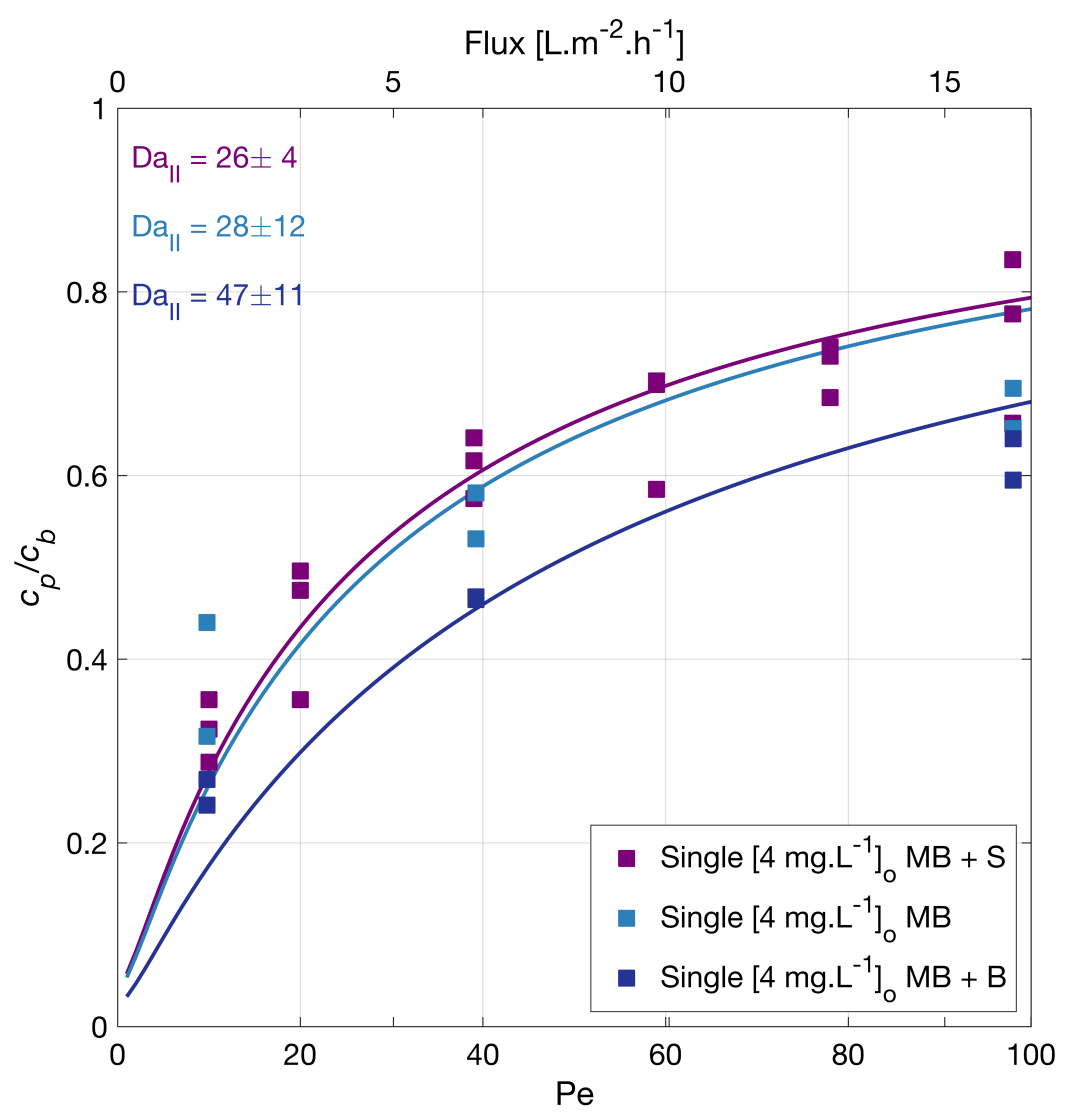
Methylene blue (MB) degradation with different backgrounds vs. filtration rate. Where the MP was alone, or with a background S (142 mg·L${}^{-1}$Na_2_SO_4_) or a background B (23.4 mg·L${}^{-1}$NaHCO_3_). Symbols depict experimental data of the MB independently, and the lines correspond to the mass transport and surface reaction model. The legend shows the feed concentrations [$c_{b}$]${}_{o}$. Conditions: 366 nm radiation, 210 W·m${}^{-2}$.

This information does not explain the degradation order observed in our experiments. In particular, MB is the compound that degrades most slowly. To rule out a scavenging effect of the sulfate groups, the degradation of MB without sodium sulfate was investigated (see Figure 2), and the degradation rate of MB overlapped with the previous, showing no inhibition effect by sulfate groups. These results also suggested no adsorption competition on the membrane surface by the counter ion in the salt, Na^+^.

The degradation of MB in the presence of sodium bicarbonate [23.4 mg·L${}^{-1}$] was also tested (Figure 2), and a slightly improved degradation upon the bicarbonate addition was found. The solution pH was 7.10 for the MB solution, 6.37 for MB with sodium sulfate, and 7.4 for MB with sodium bicarbonate. Guillard et al. [44] investigated the effect of different salts (20 mM initial concentration) in the photocatalytic MB degradation. Their findings suggest an inhibiting effect due to the deposition of salts on the TiO_2_ surface and that, at neutral and alkaline pH, the main factor affecting the MB degradation was the amount of MB adsorbed. This adsorption changes with the surface density of anionic sites, TiO^−^, available. Our test agrees with these findings, as a slight change in the pH could make the surface slightly more negative and improve the MB adsorption; hence its degradation.

### 3.3. Effect of the Water Matrix on the Photocatalytic Degradation of MPs in a Mixture

Natural waters commonly contain inorganic salts as well as other organic matters. The overall water quality has an influence on the degradation kinetics of the MPs, and in this section, various water matrices are studied.

#### 3.3.1. MPs Mixture Degradation

**Figure 3:**
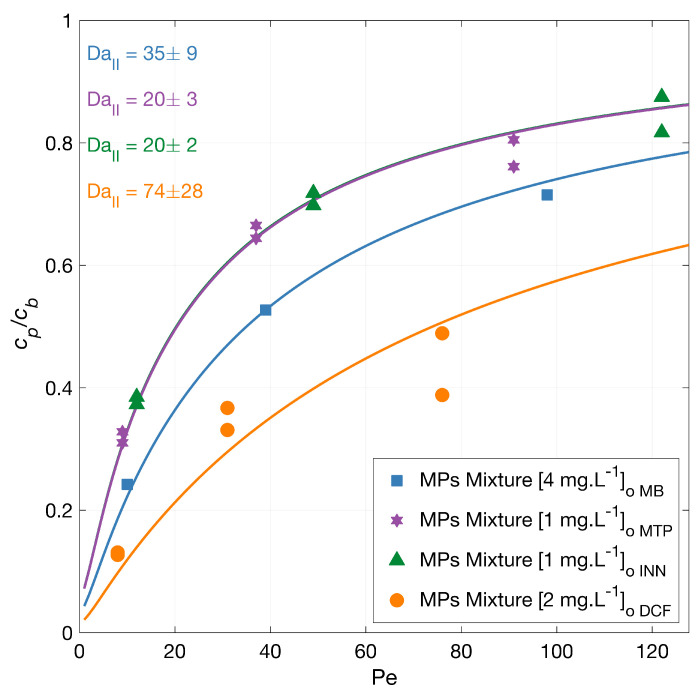
MPs mixture degradation vs. filtration rate. Symbols depict experimental data of the MPs in a mixture, and the lines correspond to the mass transport and surface reaction model. The experiments were carried out with the MPs in a mixture,. The legend shows the feed concentrations [$c_{b}$]${}_{o}$. Conditions: 366 nm radiation, 210 W·m${}^{-2}$ and 142 mg·L${}^{-1}$ Na_2_SO_4_.

The degradation of a mixture of MPs was investigated with feed solution concentration equal to the experiments using single MPs. A generally lower degradation rate is expected due to the competitive effect of the present pollutants on the generated radicals, as the total micropollutants amount is higher than in the experiments with the individual MPs. Figure 3 illustrates the normalized permeate concentration as a function of the Péclet number with the corresponding model fits. The overall micropollutants’ degradation from higher to lower varied from the experiments using singles to DCF > MB > MTP > INN, with an overlapping Damköhler value for INN and MTP. At the lower flux, the total degradation was 87% for DCF, 62% for INN, 76% for MB, and 68% for MTP. Note that the MB degradation measurement in the mixture was carried out only on one of the repetitions.

As anticipated, the degradation of most of the MPs was less in the mixture (i.e., had a higher permeate concentration) compared to the degradation experiments in singles, indicating a competition for the ROS and a competitive adsorption of the MPs on the TiO_2_ surface. Unexpectedly, the degradation of MB was similar in the mixture compared to the single measurement (Figure 1), where other MPs were less degraded.

#### 3.3.2. Effect of Bicarbonate

**Figure 4:**
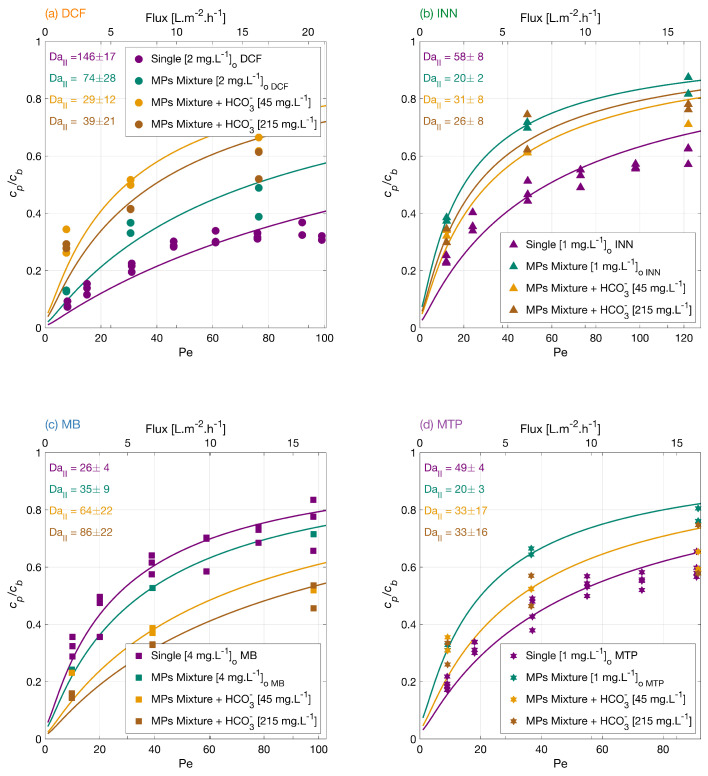
Degradation of (**a**) DCF, (**b**) INN, (**c**) MB, and (**d**) MTP, independently, in a mixture, and in a mixture with two concentrations of bicarbonate ions. Symbols depict experimental results, and lines correspond to the mass transport and surface reaction model. Conditions: the legend shows the feed concentrations [$c_{b}$]${}_{o}$, 366 nm radiation, 210 W·m${}^{-2}$, and 142 mg·L${}^{-1}$ Na_2_SO_4_.

Carbonate ions are present in aerated water and may also be formed as reaction products in the degradation of organic compounds [45]. Therefore, the effect of bicarbonate concentration on the micropollutants mixture degradation was investigated. Figure 4 shows the normalized permeate concentration of each MP in a separate plot as a function of the normalized filtration rate, Pe. The MPs degradation from higher to lower was MB > MTP > INN > DCF in the mixture with a lower concentration of bicarbonate, and the degradation amount at the lower flux was 70% for DCF, 67% for INN, 81% for MB, and 67% for MTP. Meanwhile, in the mixture with a higher concentration of bicarbonate, the order from higher to lower was MB > DCF > MTP > INN with an amount of total degradation of 71% for DCF, 68% for INN, 85% for MB, and 70% for MTP. Clearly, in these experiments, the effect of the added bicarbonate is specific for each micropollutant.

Different theories have been proposed in the literature regarding the bicarbonate effect on the photocatalytic degradation of micropollutants. There is a general consensus about the detrimental effect of bicarbonate molecules acting as scavengers of hydroxyl radicals and reducing the amount of available ROS in the system. Conversely, carbonate species can also act as conduction band electron quenchers [46], which decreases the electron-hole recombination and generates a positive impact on the photocatalytic degradation of organic molecules. At the same time, the bicarbonate ions could react with the hydroxyl radicals to generate carbonate radicals (HCO_3_^•^ and CO_3_^•^−) as oxidation transients [47] that could mediate the degradation of the MPs. However, their reactions are typically slower than those of OH radicals and with high selectivity toward organic compounds (with second-order rate constants ranging between 10${}^{2}$ and 10${}^{9}$ L·mol${}^{-1}$·s${}^{-1}$) [48,49]. Ye et al. [50] reported a positive effect with the bicarbonate addition during the photocatalytic degradation of MTP with nanotube arrays, which was related to the bicarbonate electron-quenching capability and carbonate radical mediation during the degradation reactions.

The pH in the feed solution increases upon the addition of the bicarbonate (i.e., 7.8 for the lower bicarbonate content and 8.0 for higher bicarbonate content), which may change the electrostatic interactions of the MPs to the TiO_2_ surface. The surface of the TiO_2_ membrane becomes more negative as the pH of the solution increases; thus, the surface attracts the positively charged molecules like MB and rejects the negatively charged ones like DCF. This is visible in the experimental results, as, for DCF, adding bicarbonate reduced its degradation significantly, whereas for MB, its degradation improved. For INN and MTP, the bicarbonate effect is less significant, but the degradation is improved compared with the mixture without bicarbonate.

#### 3.3.3. Effect of Chloride

**Figure 5:**
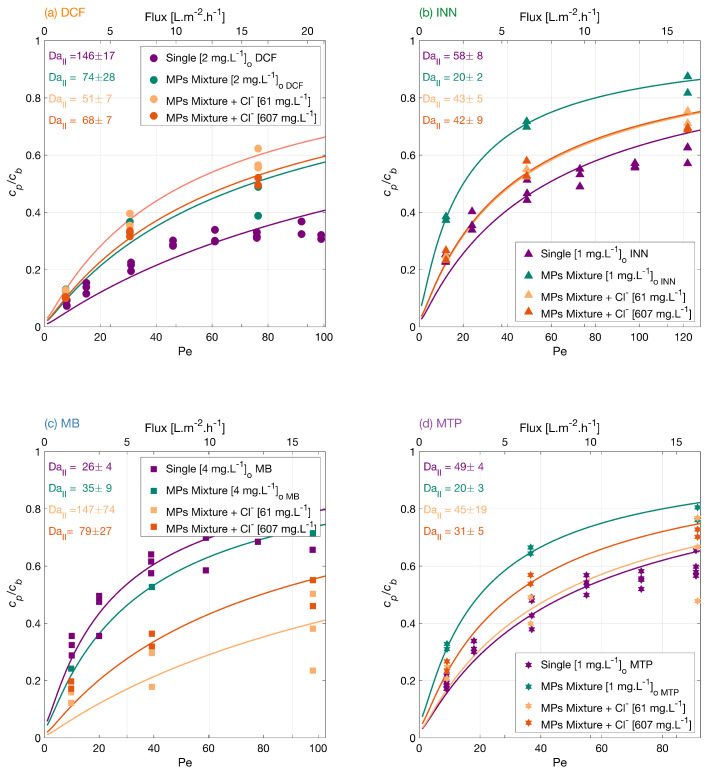
Degradation of (**a**) DCF, (**b**) INN, (**c**) MB, and (**d**) MTP independently, in a mixture and in a mixture with two concentrations of chloride ions. Symbols depict experimental results, and lines correspond to the mass transport and surface reaction model. Conditions: the legend shows the feed concentrations [$c_{b}$]${}_{o}$, 366 nm radiation, 210 W·m${}^{-2}$ and 142 mg·L${}^{-1}$Na_2_SO_4_ for the experiments with the MPs independently and in a mixture, and 23.4 mg·L${}^{-1}$ NaHCO_3_ for the experiments with sodium chloride.

Chloride ions are present in natural water. Therefore, the effect of chloride in the mixture of MPs was investigated. Figure 5 shows how the MP degradation varied when adding the different concentrations. With lower chloride concentration from higher to lower, the degradation order was MB > DCF > MTP > INN, and the degradation degree at the lower flux was 88% for DCF, 76% for INN, 86% for MB, and 77% for MTP. Meanwhile, in the mixture with a higher chloride concentration, the order changed to MB > DCF > INN > MTP, and the degradation degree at the lower flux was 90% for DCF, 75% for INN, 82% for MB, and 75% for MTP.

Chloride ions potentially scavenge the photogenerated holes and oxidize to chloride radicals. These formed radicals could back-react with the conduction band electrons, which lowers the concentration of available holes and electrons [51] and reduces recombination. However, a large amount of chloride may inhibit the generation of hydroxyl radicals as the concentration of available holes is reduced. Thus, there is a stronger improvement in the degradation of MB and MTP with 61 mg·L${}^{-1}$ of Cl^−^ than with 607 mg·L${}^{-1}$. Aguedach et al. [52] studied the ion strength effect on the degradation of a reactive black 5 azo dye, and reported an increase in the initial degradation rate and a decrease in the time needed to bleach the solution upon addition of Cl^−^ salts. They explained this effect by the improved dye adsorption on the TiO_2_ surface with the addition of the salt. Lair et al. [53], also showed a faster initial degradation of naphthalene upon NaCl addition as a result of an enhancement in the naphthalene adsorption.

For these experiments, a concentration of 23.4 mg·L${}^{-1}$ of sodium bicarbonate was used as a background, which increased the feed solution pH to 7.5 for the mixture with lower chloride concentration and to 7.3 for the mixture with a higher chloride concentration. As seen in the previous section, a higher pH hinders the photocatalytic degradation of DCF, which could explain why its degradation is lower in this water matrix.

#### 3.3.4. MPs Degradation at Low Concentration in a Mixture and the Effect of Tap Water

**Figure 6:**
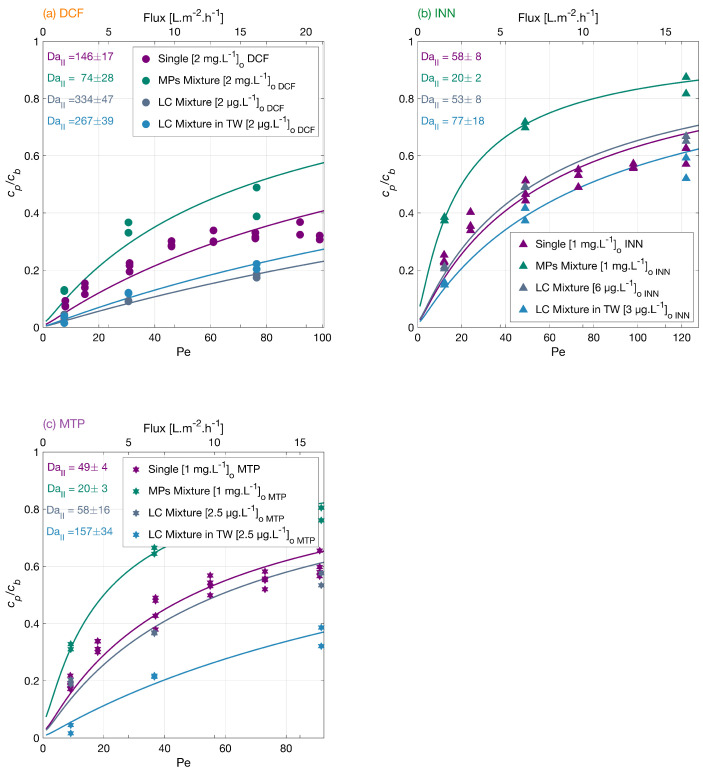
Degradation of (**a**) DCF, (**b**) INN, and (**c**) MTP independently, in a mixture, in a mixture with low concentrations and in tap water. Symbols depict experimental results, and lines correspond to the mass transport and surface reaction model. Conditions: the legend shows the feed concentrations [$c_{b}$]${}_{o}$, 366 nm radiation, 210 W·m${}^{-2}$ and 142 mg·L${}^{-1}$Na_2_SO_4_ for the experiments with the MPs independently and in a mixture, 23.4 mg·L${}^{-1}$ NaHCO_3_ for the experiments in a mixture in low concentration.

Micropollutants are found in surface water up to μg·L${}^{-1}$ levels. Therefore, it is important to study the MPs degradation at environmentally relevant concentrations and conditions. The degradation of the MP mixture at μg·L${}^{-1}$ concentrations was investigated. Figure 6 shows an order in the overall low concentration mixture degradation from higher to lower DCF > MTP > INN. The degradation degree at the lower flux was 96% for DCF, 79% for INN, and 80% for MTP, which is higher than the MPs mixture in mg·L${}^{-1}$, because the total amount of organic molecules is lower, and there is less competition for the reactive oxygen species. In the mixture in low concentration with tap water, the order in the overall micropollutants degradation from higher to lower was DCF > MTP > INN, and the degradation amount at the lower flux was 97% for DCF, 85% for INN, and 97% for MTP.

For conventional AOPs, tap water inhibits typical organic molecules degradation as there are many ions that scavenge the ROS. In our experiments, the matrix with tap water enhanced most of the MPs degradation. The degradation of DFC was significantly improved at low MPs concentration, but it was lower in TW. This difference could be explained by the pH increase from 7.3 to 8 from the MPs mixture in low concentration to the mixture in tap water. At higher pH, the membrane repulsion toward the negatively charged DCF increases.

### 3.4. Comparison of the Photocatalytic Degradation Rate with Different Water Matrices

Eight different water matrices were studied to evaluate the MPs degradation by a photocatalytic membrane in a PMR with fluxes in the nanofiltration rate. The results, achieved in a single-pass operation, showed the capability of the system to degrade pharmaceuticals under all the studied water matrices. Table 3 shows the surface reaction rate constant for the degradation of DCF, INN, MB, and MTP under the studied water matrices. The surface reaction rate constant $k^{\prime }$ [m·s${}^{-1}$] relates to the fitted second Damköhler number by Da${}_{II}\;=\;k^{\prime }L/D$, where *L* represents the height of the liquid reservoir [m] and *D* the compounds diffusivity [m${}^{2}$·s${}^{-1}$]. When comparing all the water matrices, relatively, the most degradation was obtained with the MPs in low concentration and in tap water.

Most of the phenomena related to the addition of ions during photocatalytic processes are explained by the recombination effect, although there is little known about when and how the recombination occurs [54]. An increase in pH resulted in an improved degradation for the positively charged MPs (INN, MB, and MTP). The pH potentially enhances the negative surface charge of the membrane and, with that, the adsorption of positively charged contaminants. Surface redox reactions are generally more efficient when species are pre-adsorbed [8]. Arlos et al. [13] reported this effect in a study with a negative and a positively charged photocatalytic membrane with mixtures of molecules with cationic and anionic groups.

The effect of added salts during the experiments with photocatalytic membranes enhanced, in general, the reaction in accordance with other experiments with salts and immobilized TiO_2_ [50,52]. These results show that immobilized TiO_2_ systems have a significant advantage over conventional TiO_2_ slurry systems where the presence of ions hinders the photocatalytic degradation of organic molecules [44,51,55,56].

## 4. Conclusions

Photocatalytic membranes in a flow-through single-pass photocatalytic membrane reactor were investigated for the elimination of MPs in various water matrices. The degradation rates of diclofenac, iopamidol, methylene blue, and metoprolol varied among the compounds and water matrices. The presence of anions such as bicarbonate, usually reported as a degradation inhibitor, positively impacts the degradation efficiency of the positively charged MPs, suggesting the importance of the surface charge interactions between the MP and the photocatalytic surface. The presence of chloride also contributed positively to the MPs degradation, more significantly in low concentrations than in high concentrations. Chloride ions can potentially scavenge photogenerated holes preventing electron-hole recombination, but at high chloride concentrations, the amount of available hydroxyl radicals is reduced with the number of available holes. The results of mixtures at environmentally relevant concentrations also showed surprising results, revealing an improved degradation of MPs in tap water with degradation of 97% for DCF, 85% for INN, and 97% for MTP. The findings from this lab-scale study have provided further insights into the applicability of photocatalytic membranes for micropollutants degradation processes.

## Figures and Tables

**Table 1:** Diffusion coefficient.

Micropollutant	D${}_{t}$ [10${}^{-10}$ m${}^{2}$·s${}^{-1}$]	D${}_{e}$ [10${}^{-10}$ m${}^{2}$·s${}^{-1}$]
Diclofenac	5.90 [29]	5.28
Iopamidol	-	3.70
Methylene Blue	4.60 [30]	5.40
Metoprolol	-	4.93

**Table 3:** Surface reaction rate constant, $k^{\prime }$ [10${}^{-6}$ m·s${}^{-1}$].

Solution	DCF	INN	MB	MTP
DCF + S	9 ± 1	-	-	-
INN + S	-	2.2 ± 0.3	-	-
MB	-	-	1.3 ± 0.5	-
MB + S	-	-	1.2 ± 0.2	-
MB + B	-	-	2.2 ± 0.5	-
MTP + S	-	-	-	2.4 ± 0.2
MPs mixture + S	4 ± 2	0.8 ± 0.1	1.6 ± 0.4	1.0 ± 0.2
MPs mixture + S + HCO_3_^−^ 45 mg·L${}^{-1}$	1.7 ± 0.7	1.1 ± 0.3	3 ± 1	1.6 ± 0.9
MPs mixture + S + HCO_3_^−^ 215 mg·L${}^{-1}$	2 ± 1	1.0 ± 0.3	4 ± 1	1.6 ± 0.8
MPs mixture + B + Cl^−^ 61 mg·L${}^{-1}$	3.0 ± 0.4	1.6 ± 0.2	7 ± 3	2 ± 1
MPs mixture + B + Cl^−^ 607 mg·L${}^{-1}$	4.0 ± 0.4	1.6 ± 0.3	4 ± 1	1.5 ± 0.2
MPs LC mixture + B	20 ± 3	1.9 ± 0.3	-	2.9 ± 0.8
MPs LC mixture in tap water	16 ± 2	2.9 ± 0.7	-	8 ± 2

Reaction conditions: 366 nm radiation, 210 W.m^−2^ and background salt (S = 142 mg·L^−1^ Na2SO4 and B = 23.4 mg·L^−1^ NaHCO_3_). MPs mixture refers to experiments done with micropollutants mixtures. LC refers to low (environmentally realistic) concentrations for the micropollutants in the mixture.

**Figure A1:**
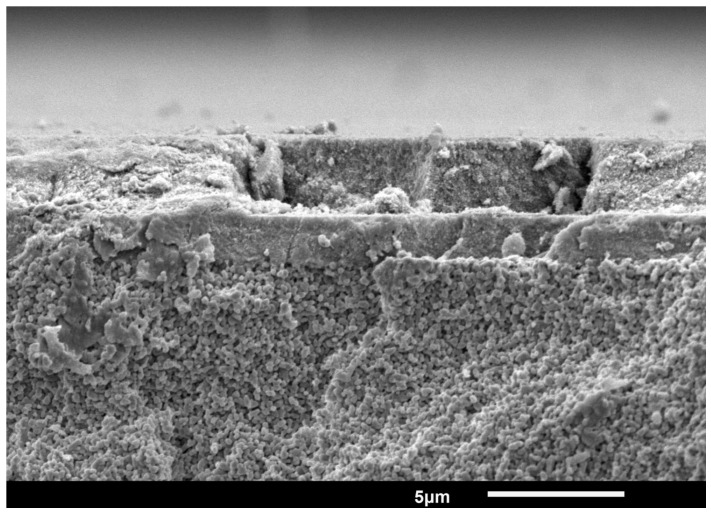
SEM cross-sectional picture of titania and γ-alumina layers on an α-alumina support.

**Figure A2:**
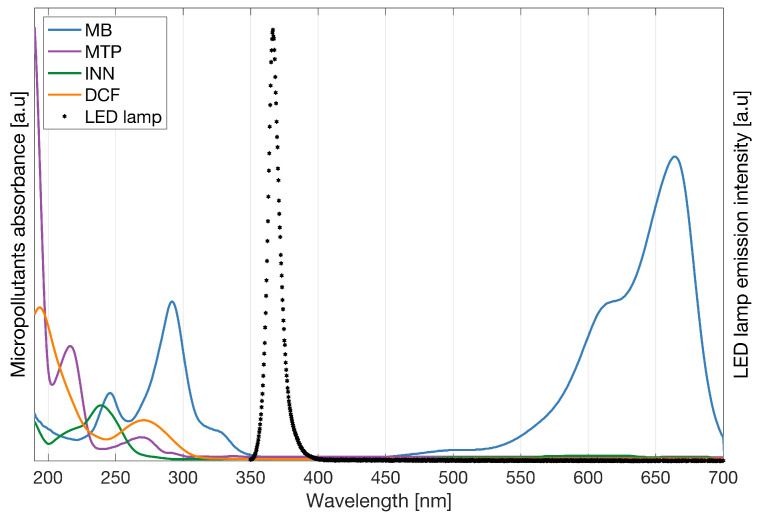
Combined spectrum of the micropollutants absorbance and LED lamp emission intensity.

## Abbreviations

The following abbreviations are used in this manuscript:

AOP	Advanced Oxidation Process
B	Sodium Bicarbonate in the background [23.4 mg·L${}^{-1}$]
DCF	Diclorofenac
INN	Iopamidol
MB	Methylene Blue
MPs	Micropollutants
MTP	Metoprolol
LC	Low concentration
PMR	Photocatalytic Membrane Reactor
pzc	Point of zero charge
ROS	Reactive oxygen species
TW	Tap water

## Appendix A. Tap Water Composition

Tap water used for the experiments was sampled from the laboratory at Wetsus (Leeuwarden, The Netherlands). The chemical composition of the tap water is described in Table A1.

**Table A1:** Chemical composition of the MPs mixture in tap water.

Parameter	Unit	Stock Solution
Cl	mg·L${}^{-1}$	46.2
NO_2_	mg·L${}^{-1}$	<0.10
NO_3_	mg·L${}^{-1}$	10.4
PO_4_	mg·L${}^{-1}$	<0.10
SO_4_	mg·L${}^{-1}$	0.58
TC	mg·L${}^{-1}$	65.3
NPOC	mg·L${}^{-1}$	3.83
IC	mg·L${}^{-1}$	48.6
Ca	μg·L${}^{-1}$	33,700
Cu	μg·L${}^{-1}$	14.3
Ca	μg·L${}^{-1}$	33,700
Fe	μg·L${}^{-1}$	<5.00
K	μg·L${}^{-1}$	2330
Mg	μg·L${}^{-1}$	9343
Na	μg·L${}^{-1}$	69,550

TC = Total Carbon, NPOC = Non-Purgeable Organic Carbon, IC = Inorganic Carbon.

## Appendix B. Feed and Permeate pH

The pH of the solution was measured for the feed solutions and the permeate and is presented in Table A2.

**Table A2:** Measured pH.

Solution	pH${}_{b}$	pH${}_{p}$
DCF + S	6.6 ± 0.2	6.6 ± 0.9
INN + S	6.97 ± 0.02	6.2 ± 0.3
MB	7.10 ± 0.06	-
MB + S	6.37 ± 0.06	6.49 ± 0.04
MB + B	7.4 ± 0.1	7.1 ± 0.1
MTP	6.47 ± 0.08	6.0 ± 0.3
Na_2_SO_4_	5.91 ± 0.05	-
MPs mixture + S	6.2 ± 0.2	6.89 ± 0.08
MPs mixture + S + HCO_3_^−^ 45 mg·L${}^{-1}$	7.8 ± 0.1	7.9 ± 0.07
MPs mixture + S + HCO_3_^−^ 215 mg·L${}^{-1}$	8.00 ± 0.03	8.3 ± 0.1
MPs mixture + B + Cl^−^ 61 mg·L${}^{-1}$	7.54 ± 0.09	7.5 ± 0.3
MPs mixture + B + Cl^−^ 607 mg·L${}^{-1}$	7.3 ± 0.1	6.5 ± 0.1
MPs LC mixture + B	7.24 ± 0.02	7.4 ± 0.2
MPs LC mixture in tap water	8.01 ± 0.04	8.23 ± 0.01

pH_*b*_ = feed pH, pH_*p*_ = permeate pH, average of the tested flows, and background salts: S = 142 mg·L^−1^ Na_2_SO_4_ and B = 23.4 mg·L^−1^ NaHCO_3_.

## Appendix C. Membranes Characterization

For the characterization of the membrane, high resolution SEM (Analysis Zeiss MERLIN HR-SEM) was used to investigate the morphology and cross-section. The layers of membrane A have an average thickness of 1.2 μm for the gamma layer and 3.2 μm for the TiO_2_ layer and for membrane B of 1.6 μm for the gamma layer and 2.8 μm for the TiO_2_ layer. Figure A1 depicts an SEM image of the photocatalytic membrane B.

The pore size of the membranes was determined by permporometry using cyclohexane as condensing vapor [57]. The pore size characterization showed 5 ± 0.1 nm mesopores for both membranes, which corresponds to the pore size of the γ-alumina layers.

These membranes can be cleaned by calcination and reused, which is an important factor in their implementation for real-world applications. Information on the fabrication of the membrane can be found elsewhere [16].

## Appendix D. Absorption Spectrum

The absorption of light by the micropollutants was investigated from wavelengths between 200 and 700 nm, see Figure A2. None of the micropollutants in the study absorbs photons at the wavelength used during the experiment ($\lambda_{max}$ = 366 nm).

To protect the micropollutants from any reaction with the ambient light, the dead-end filtration and degradation experiments were performed inside a cupboard, and all of the solutions were stored in opaque bottles with the extra protection of aluminum paper around them.
